# RETRACTION: Comprehensive Analysis of Mango (*Mangifera indica* L.) Seed: Phytochemical Profile, Bioactivity, and Nutraceutical Potential

**DOI:** 10.1002/fsn3.71412

**Published:** 2026-01-19

## Abstract

**RETRACTION**: ImanS. , . ur RasheedM , ZahranH. A. , RashidH. , FatimaM. , SaleemZ. , BanoY. , GullS. , AkbarR. , KhanA. , AhmedI. A. M. , ZongoE. , RahimM. A. , and Castro‐MuñozR. , "Comprehensive Analysis of Mango (*Mangifera indica* L.) Seed: Phytochemical Profile, Bioactivity, and Nutraceutical Potential", Food Science & Nutrition 13, no. 6 (2025): e70390. 10.1002/fsn3.70390.40524682 PMC12168231

The above article, published online on 16 June 2025 in Wiley Online Library (wileyonlinelibrary.com), has been retracted by agreement between the journal Editor‐in‐Chief, Y. Martin Lo; and Wiley Periodicals LLC. A third party reported that overlapping panels were detected between several images in Figure 7 which are meant to report on different tissue samples: 7A and 7B, 7C and 7D, 7G and 7H, and 7I and 7J. Additionally, Figure 3 included several trace and text distortions; distortions and duplicated peaks were detected in Figure 5; and the graphical text had been distorted in all images in Figure 6. Lastly, a modified version of Figure 5 has been published in another article by some of the same authors [Rasheed et al. 2025 (https://doi.org/10.1186/s12870‐025‐06997‐7)]. Both articles were under review by their respective journals at the same time.

The author responded to an inquiry by the publisher and confirmed that the distortions in the figures were due to using software to upscale Figures 5 and 6. Regarding the images duplicated across publications, the authors stated that that the topics of the publications were related and as such the same data were published in both articles. The authors denied that multiple tissue samples in Figure 7 were overlapping and stated that the similarities might be due to the uniform histological architecture of the tissues.

This retraction has been agreed to because of the number of errors in the figures, the instances of data shared across two publications, and the overlapping tissue sections in Figure 7, concerns which fundamentally compromise the editor's confidence in the results presented. The authors did not respond to our notice regarding the retraction.

**RETRACTION**: S. Iman , M. ur Rasheed , H. A. Zahran , H. Rashid , M. Fatima , Z. Saleem , Y. Bano , S. Gull , R. Akbar , A. Khan , I. A. M. Ahmed , E. Zongo , M. A. Rahim , and R. Castro‐Muñoz , "Comprehensive Analysis of Mango (*Mangifera indica* L.) Seed: Phytochemical Profile, Bioactivity, and Nutraceutical Potential", Food Science & Nutrition 13, no. 6 (2025): e70390. 10.1002/fsn3.70390.40524682 PMC12168231

The above article, published online on 16 June 2025 in Wiley Online Library (wileyonlinelibrary.com), has been retracted by agreement between the journal Editor‐in‐Chief, Y. Martin Lo; and Wiley Periodicals LLC. A third party reported that overlapping panels were detected between several images in Figure 7 which are meant to report on different tissue samples: 7A and 7B, 7C and 7D, 7G and 7H, and 7I and 7J. Additionally, Figure 3 included several trace and text distortions; distortions and duplicated peaks were detected in Figure 5; and the graphical text had been distorted in all images in Figure 6. Lastly, a modified version of Figure 5 has been published in another article by some of the same authors [Rasheed et al. 2025 (https://doi.org/10.1186/s12870‐025‐06997‐7)]. Both articles were under review by their respective journals at the same time.

The author responded to an inquiry by the publisher and confirmed that the distortions in the figures were due to using software to upscale Figures 5 and 6. Regarding the images duplicated across publications, the authors stated that that the topics of the publications were related and as such the same data were published in both articles. The authors denied that multiple tissue samples in Figure 7 were overlapping and stated that the similarities might be due to the uniform histological architecture of the tissues.

This retraction has been agreed to because of the number of errors in the figures, the instances of data shared across two publications, and the overlapping tissue sections in Figure 7, concerns which fundamentally compromise the editor's confidence in the results presented. The authors did not respond to our notice regarding the retraction.

